# The Impact of a Dental Technology Curriculum and a Preventive-Clinical Curriculum on the Development of Preclinical Preparation Skills

**DOI:** 10.3390/dj14080475

**Published:** 2026-08-03

**Authors:** Lisa Morlock, Marius Morlock, Ann Katrin Reuter, Thekla J. Pfeiffer-Grötz, James Deschner

**Affiliations:** 1 Department of Periodontology and Operative Dentistry, University Medical Center, Johannes Gutenberg University, 55131 Mainz, Germany; 2 Private Practice–ZahnZuliebe, Schillerstraße 24, 55116 Mainz, Germany; 3 Research Center for Immunotherapy (FZI), Johannes Gutenberg University, 55131 Mainz, Germany

**Keywords:** fine motor skills, preclinical dental curriculum, dental education, simulation course, preparation performance

## Abstract

**Background/Objectives:** Fine motor skills are essential for procedural precision in dental education. This study evaluated preparation performance and self-assessed skills among students completing either a dental technology and materials science-based curriculum (DTMS) or a shorter prevention-oriented curriculum (PREV). Assessments were conducted before and after a shared preclinical simulation course. We hypothesized that DTMS students would perform better at baseline and that differences would diminish after the course. **Methods**: Students in the DTMS and PREV groups prepared a standardized practice block at the beginning and end of a 14-week shared simulation course. Preparations were evaluated for depth, form, and surface smoothness. Students also completed a self-assessment questionnaire using a five-point Likert scale. **Results**: Sixty-four students participated (68.8% female). At baseline, the PREV group (PREVG) achieved significantly higher overall scores (*p* = 0.036) and better adherence to preparation form (*p* = 0.011) than the DTMS group (DTMSG). Following the simulation course, the PREVG showed significant improvements in overall scores (*p* = 0.009), preparation form (*p* < 0.001), and surface smoothness (*p* < 0.001), accompanied by improved self-assessed preparation skills (*p* = 0.006). In contrast, the DTMSG showed no significant changes in overall scores or individual quality parameters (*p* > 0.05). **Conclusions**: A prevention-oriented preclinical curriculum focusing on structured dental procedures was not inferior to a more extensive materials science-based curriculum despite fewer practical exercises. Continuous practice remains essential, while reduced practical workloads may provide curricular capacity for modern interdisciplinary teaching.

## 1. Introduction

Practical skills are essential for the dental profession. More than 30 years ago, there were calls internationally for a reform of dental education that would take account of developments and advances in dentistry [1]. Globally, dental education is undergoing a transformation to take account of technical innovations and advances [2,3,4].

The preclinical phase lays the groundwork for the subsequent clinical phase, which enables the treatment of patients. The focus here is on developing manual dexterity and forming a professional identity [5]. There is a correlation between students’ preclinical and clinical performance, which highlights the importance of effective preclinical training [6].

Preclinical teaching usually takes place in a simulated environment, such as using a phantom head simulator, in order to bridge the gap between the preclinical and clinical curriculum and enable realistic training [7,8].

Fine motor skills correlate with students’ clinical practical skills and can be improved through targeted exercises [9]. The study by Pérez-Chicharro et al. shows that even short-term training programs lead to significant improvements in the manual dexterity of dental students. However, this is dependent on them participating in targeted training programs and, in particular, practicing continuously [10].

Nevertheless, the manual skills required for the intraoral preparation of teeth remain a key challenge in preclinical dental training [11,12].

When developing dental curricula, determining the optimal scope and structure of preclinical training remains challenging, particularly with regard to balancing theoretical knowledge and practical training in order to prepare students as effectively as possible for patient care.

The aim of this study was to compare the preparatory skills of students enrolled in a longer, predominantly dental technology and materials science-based preclinical curriculum with those of students following a shorter, predominantly preventive-oriented curriculum, both at the beginning and at the end of a shared simulation course. A better understanding of these differences may inform recommendations regarding the structure, extent, and focus of preclinical practical training.

We hypothesized that students in the dental technology and materials science (DTMS) curriculum would demonstrate superior performance at baseline, while students in the preventive (PREV) curriculum would be able to achieve comparable performance levels by the end of the course.

## 2. Materials and Methods

### 2.1. Sample Composition

The study was conducted between April 2024 and February 2025 at Johannes Gutenberg University Mainz and included third-year dental students. Participants were assigned to either a DTMS group or a PREV group. Participants were assigned to either the DTMS or PREV group according to the undergraduate curriculum in which they were enrolled. As the study was conducted during the implementation of a nationwide curriculum reform in Germany, group allocation was predetermined by the respective curriculum and randomization was not possible. Only the practical training components of the two curricula were compared in this study. In both the DTMS and PREV curricula, practical training was organized in separate courses using traditional phantom heads. These courses differed not only in the overall amount of practical training but also in their educational focus. The DTMS group (DTMSG) completed an extended preclinical curriculum with a focus on dental technology and dental materials science, comprising 840 teaching units of practical training (630 h). Most practical training was laboratory-based and focused on dental technology and materials science, while phantom-head exercises primarily emphasized prosthodontic procedures, such as full-crown preparation. In contrast, the PREV group (PREVG) followed a shorter, prevention-oriented curriculum with a reduced practical component of 317 teaching units (238 h), with the focus here being primarily on the dental treatment procedures. This practical training included preventive procedures such as professional tooth cleaning, periodontal instrumentation, clinical examination and diagnosis, and phantom-based exercises focusing on the structured sequence of clinical dental treatment procedures. The student-to-instructor ratio during practical training was identical in both curricula, providing a comparable level of supervision.

Inclusion criteria comprised enrollment in the third-year simulation course and attendance at both preparatory sessions. In addition, participants were required to complete and submit questionnaires at both assessment time points. Participation in the study was voluntary, and students received detailed information about the study procedures in advance. The study protocol was reviewed and approved by the Ethics Committee of the Rhineland-Palatinate Medical Association under application number 2024-17510.

### 2.2. Study Design

As part of the study, participants prepared a set of six specimens (Frasaco OSY preparation trainer, Frasaco GmbH, Tettnang, Germany) at baseline and at the end of a 14-week shared simulation course, during which all participants took part in the same preclinical course. In addition, students completed a questionnaire to self-assess their preparation skills at both time points.

### 2.3. Preparation Exercise

**Figure 1:**
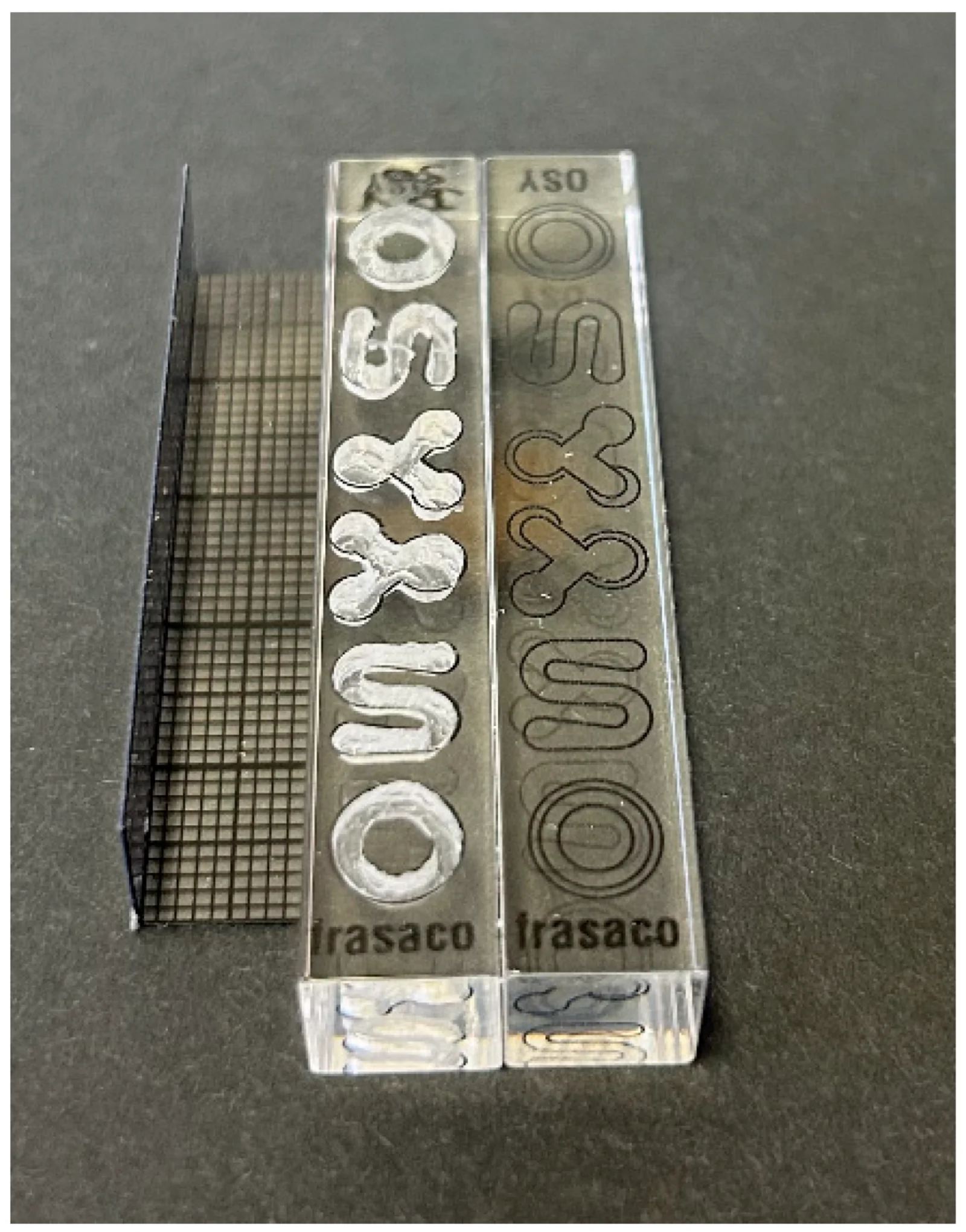
Frasaco OSY Preparation Trainer.

The students prepared the six sections using a phantom head simulator (Figure 1). The instructions were to prepare to a depth of 3 mm, extend the preparation to the outer outline, and ensure that the surface of the cavity floor was smooth. To ensure standardized working conditions, each participant received a new, identical diamond bur with an FG shank (109 010, blue; Hager & Meisinger GmbH, Neuss, Germany). Apart from the standardized task instructions and the 30 min time limit, no specific preparation protocol was prescribed, allowing participants to use their preferred preparation technique.

### 2.4. Assessment of the Preparation Exercise

The preparations were evaluated in a blinded procedure by three dentists, each with more than six years of teaching experience in preclinical dental education. Before the assessments, the examiners underwent a calibration procedure based on the approach described by Haj-Ali et al. [13]. The assessment criteria were reviewed jointly, and representative sample preparations not included in the study were evaluated and discussed until consensus on the application of the scoring criteria was achieved. To ensure blinding, all specimens were assigned unique identification numbers by the study coordinator. These codes were known only to the study coordinator, while the examiners remained blinded to both the participants’ group allocation and the assessment time point (baseline or follow-up). For each of the six specimens, three criteria were assessed: preparation depth, adherence to the prescribed preparation form, and smoothness of the cavity floor.

Each criterion was scored on a scale from 0 to 2 points according to predefined evaluation criteria (Table 1). The points awarded for all specimens and criteria were summed to obtain an overall score. The maximum achievable overall score was 36 points, calculated from six specimens assessed across three criteria, with a maximum of 2 points per criterion. Accordingly, each individual criterion (depth, preparation form, and smoothness) could reach a maximum of 12 points.

Based on the overall score, a grade on a six-point scale ranging from very good (1) to insufficient (6) was assigned. The conversion of points into grades followed the guidelines of the Institute for Medical and Pharmaceutical Examination Questions (IMPP): very good (1): 36–31.5 points; good (2): 31–27 points; satisfactory (3): 26.5–22.5 points; sufficient (4): 22–18 points; poor (5): 17.5–13.5 points; insufficient (6): <13 points.

### 2.5. Questions for Self-Assessment of Personal Preparation Skills

Data collection was carried out using pseudonymized personal codes to enable the longitudinal trends to be assessed. The questionnaire was specifically developed for this study by experienced dental educators based on the learning objectives and assessment criteria of the practical course. Prior to implementation, it was pilot-tested with eight students to assess clarity, comprehensibility, and feasibility. Participants reported that the questionnaire was easy to understand and complete, with no ambiguities or difficulties identified. The average completion time was approximately three minutes, and no modifications to the questionnaire were considered necessary.

#### 2.5.1. Sociodemographic Parameters

The following socio-demographic parameters were collected: age, gender and previous education.

#### 2.5.2. Questionnaire for Self-Assessment of Preparation Skills

The questionnaire assessing preparation performance contained four questions, which were answered using a five-point Likert scale from 1: strongly disagree to 5: strongly agree:

2.1: I am satisfied with my preparations.
2.2: I adhered to the specified depth of preparation.
2.3: I adhered to the specified preparation forms.
2.4: I have prepared the preparation trainer smoothly/even.

In addition, there was a question regarding the overall assessment of the preparation performance using a school grade from 1: very good to 6: insufficient.

### 2.6. Statistical Analysis

The statistical analysis and presentation of the data were carried out using SPSS Statistics 29 (IBM, Armonk, NY, USA) and Excel 2016 (Microsoft, Redmond, WA, USA). As this quasi-experimental study included all eligible third-year dental students during the transition between the former and revised undergraduate dental curricula, no a priori sample size calculation was performed. To evaluate the adequacy of the available sample, a sensitivity analysis was conducted using G*Power 3.1. Assuming a significance level of α = 0.05, a power of 80%, two groups, two repeated measurements, and the observed correlation between repeated measurements (r = 0.612), the available sample size (N = 64) was sufficient to detect interaction effects of Cohen’s f = 0.16 or larger. The students’ self-assessment was recorded at t0 and t1 in order to measure changes in their self-assessment of their preparation skills. Comparisons of means between the two measurement points were analyzed using the Wilcoxon signed-rank test for paired samples, as the Kolmogorov–Smirnov test indicated that the data were not normally distributed. In addition, the DTMS and PREV groups were compared using the Mann–Whitney U test. For the statistical analysis of the external assessment of the students’ preparation performance, a repeated-measures ANOVA was conducted to assess changes in preparation performance between the groups and between the two measurement time points. The mean of the assessments by the three dentists was used for further comparisons. Fleiss’ kappa was used to assess reliability. The significance level was set at *p* < 0.05 in each case.

AI-assisted translation was used for parts of this manuscript. All translated text was reviewed and approved by the authors, who take full responsibility for the final content.

## 3. Results

### 3.1. Sociodemographic Parameters

In the study, 64 third-year dental students participated. Of those students, 20 (31.25%) were male and 44 (68.75%) were female. One participant had to be excluded due to non-attendance at the second appointment, resulting in an inclusion rate of 98.46%. The average age of the participants was 25.22 years (Table 2). Twenty students were in the DMTS group and 44 students were in the PREV group.

### 3.2. Mean Values of the Total Scores

**Figure 2:**
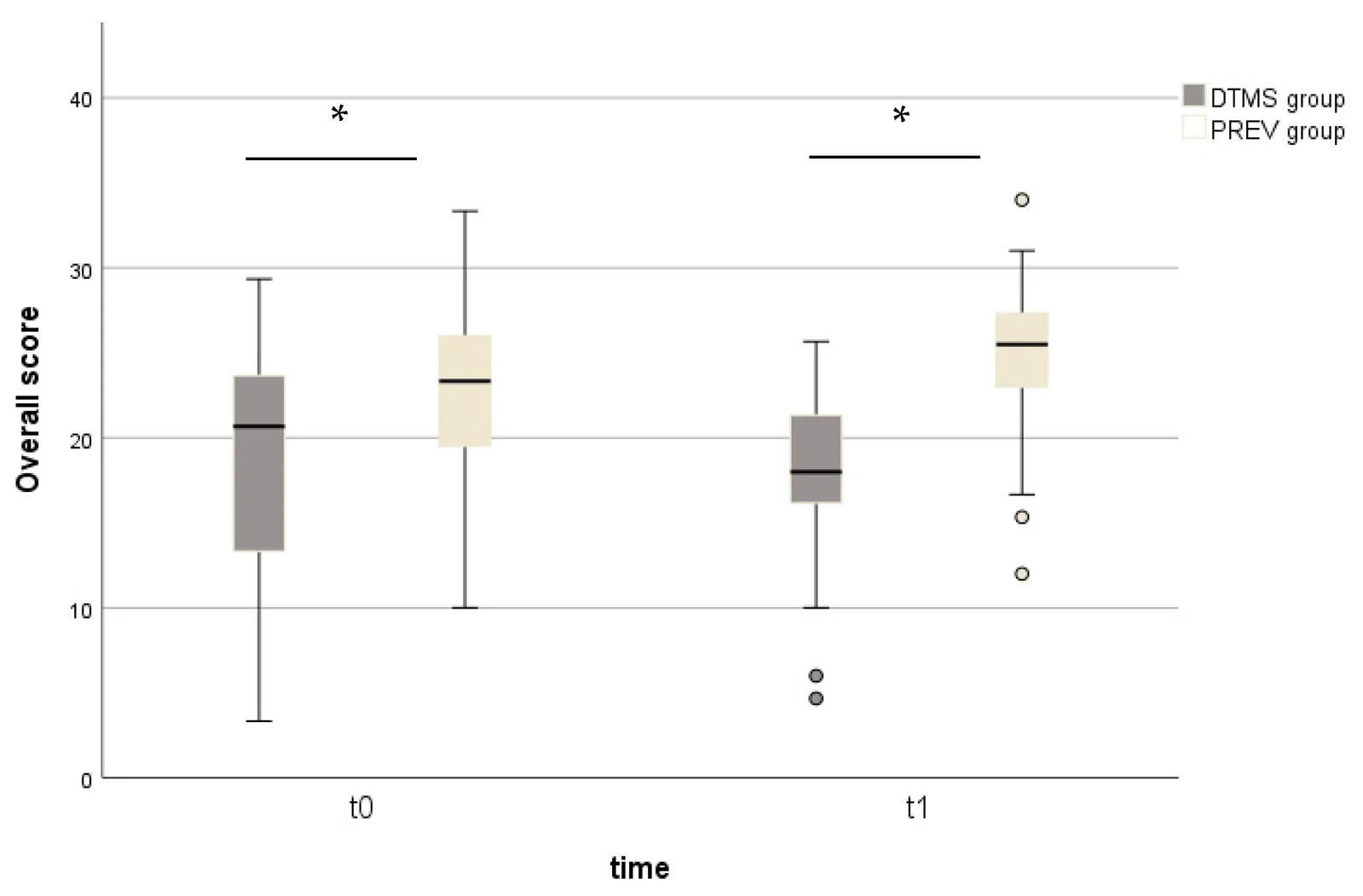
Mean values of the total scores of the DTMS and the PREV group at t0 and t1 (* significant at *p* < 0.05).

At time t0, the mean total score was 22.76 in the PREVG and 18.37 out of 36 in the DTMSG. The PREVG improved to 24.89, representing a significant improvement (*p* = 0.009), whereas in the DTMSG the value of 17.68 showed no significant change (*p* = 0.380). At both points, the total score of the PREVG was significantly higher than that of the DTMSG (t0: *p* = 0.036, t1: *p* < 0.001) (Figure 2). The interrater reliability showed a moderate agreement according to Fleiss’ kappa 0.42. However, ANOVA revealed no significant systematic differences between raters over time (*p* = 0.897), indicating a stable overall rating behavior.

### 3.3. Score by Evaluation Parameter: Depth of Preparation

**Figure 3:**
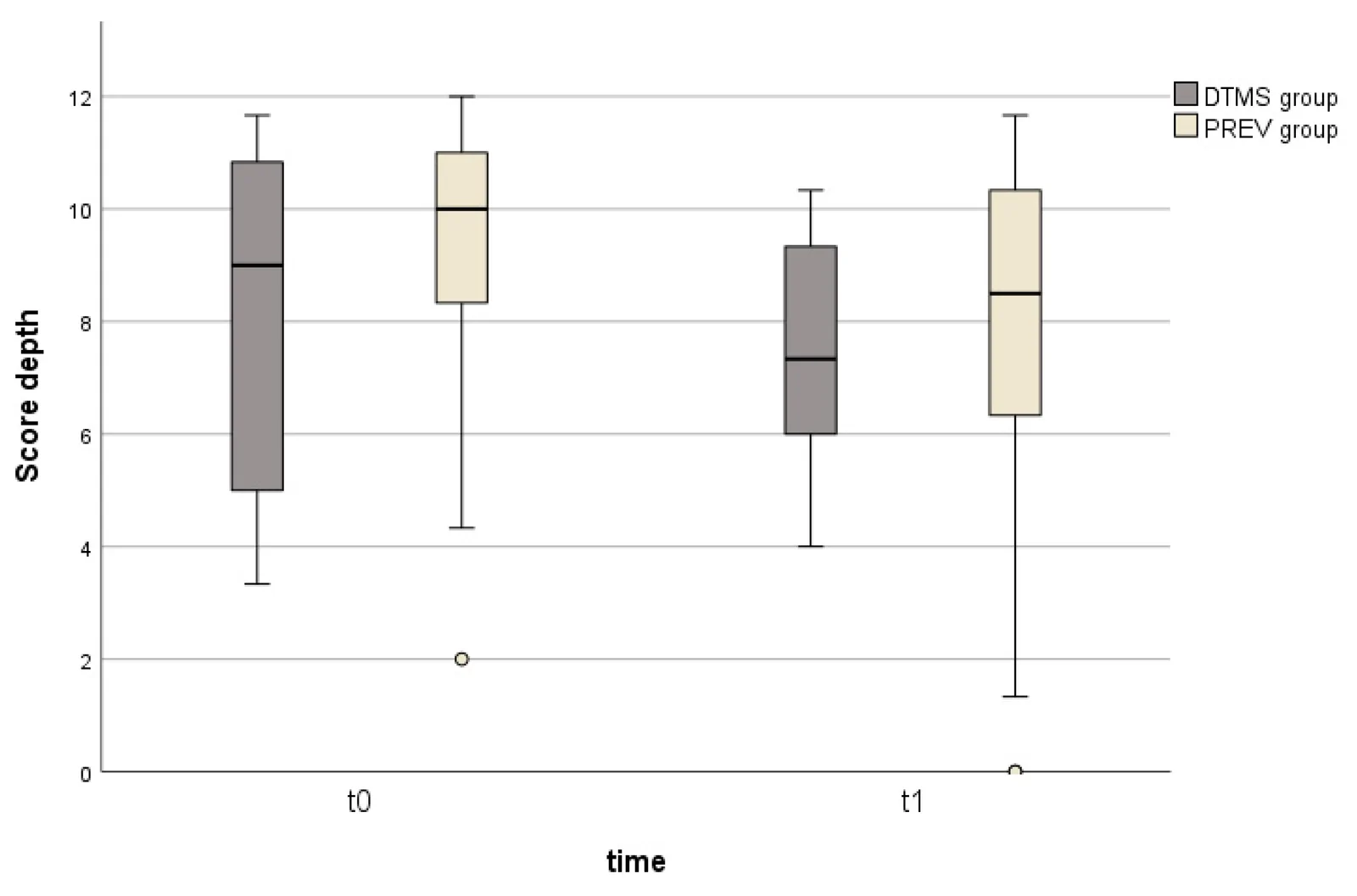
Score depth of the DTMS and the PREV group at t0 and t1.

At time point t0, the mean total preparation depth score was 9.25 in the PREVG and 8.01 out of 12 in the DTMSG. In both groups, the total preparation depth scores decreased to 8.06 in the PREVG and 7.45 in the DTMSG. The difference in the DTMSG is not significant (*p* = 0.226), whereas the PREVG shows a significant decrease in the preparation depth score (*p* = 0.007). The differences between the DTMSG and the PREVG were not significant at either measurement point (t0: *p* = 0.219, t1: *p* = 0.143) (Figure 3).

### 3.4. Score by Evaluation Parameter: Preparation Form

**Figure 4:**
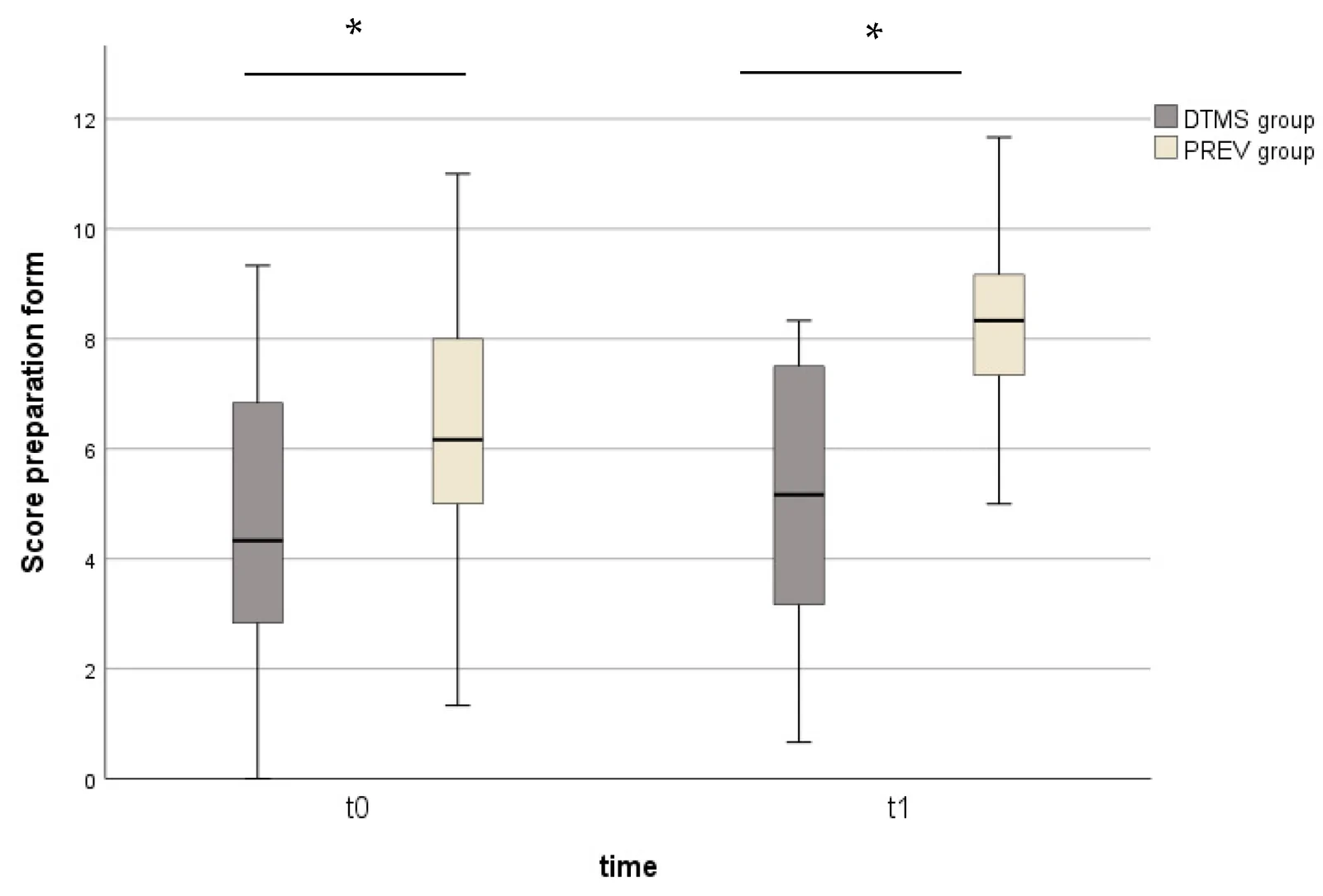
Score preparation form of the DTMS and the PREV group at t0 and t1 (* significant at *p* < 0.05).

At t0, the mean total score for the PREVG was 6.39 and for the DTMSG 4.55 out of 12. In the PREVG, the total score rose to 8.24 at t1, representing a significant improvement (*p* ≤ 0.001). In the DTMSG, the score at t1 was 5.08. This increase was not significant (*p* = 0.500). In the intergroup comparison, the PREVG performed better than the DTMSG at both time points (t0: *p* = 0.011, t1: *p* < 0.001) (Figure 4).

### 3.5. Score Based on the Evaluation Parameter Smoothness of Preparation

**Figure 5:**
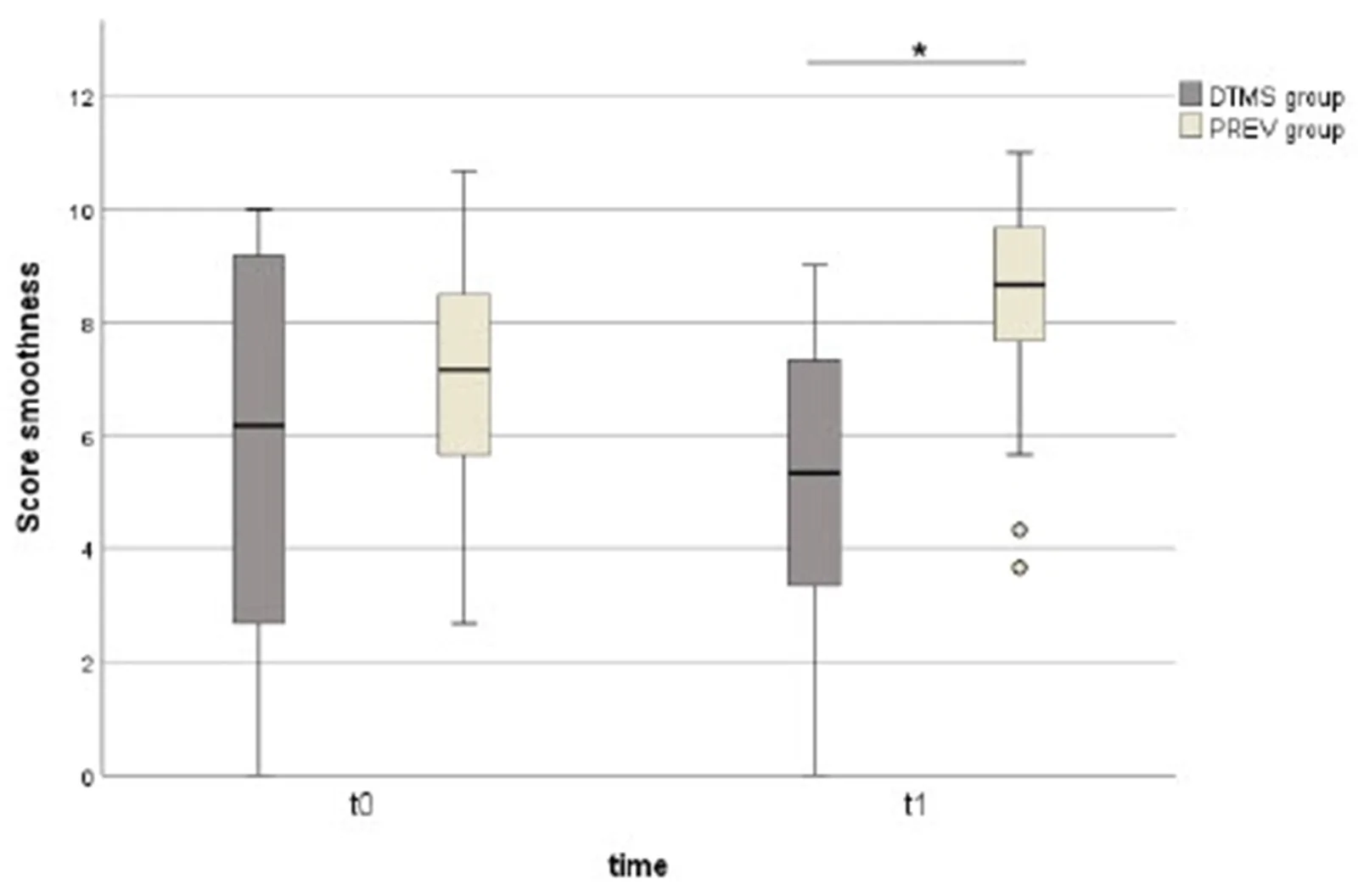
Score smoothness of the DTMS and the PREV group at t0 and t1 (* significant at *p* < 0.05).

The mean total score for assessing the smoothness of the preparation was 7.13 out of 12 in the PREVG at t0 and 5.80 in the DTMSG. At time point t1, the mean score rose to 8.53 in the PREVG and fell to 5.15 in the DTMSG. The difference in the DTMSG is not significant (*p* = 0.259), whereas the improvement in the PREVG between the two time points is significant (*p* < 0.001). There is no significant difference between the DTMSG and the PREVG at t0 (*p* = 0.197), but at t1, the PREVG shows a significantly higher value (*p* < 0.001) (Figure 5).

### 3.6. Students’ Self-Assessment of Their Preparations

**Figure 6:**
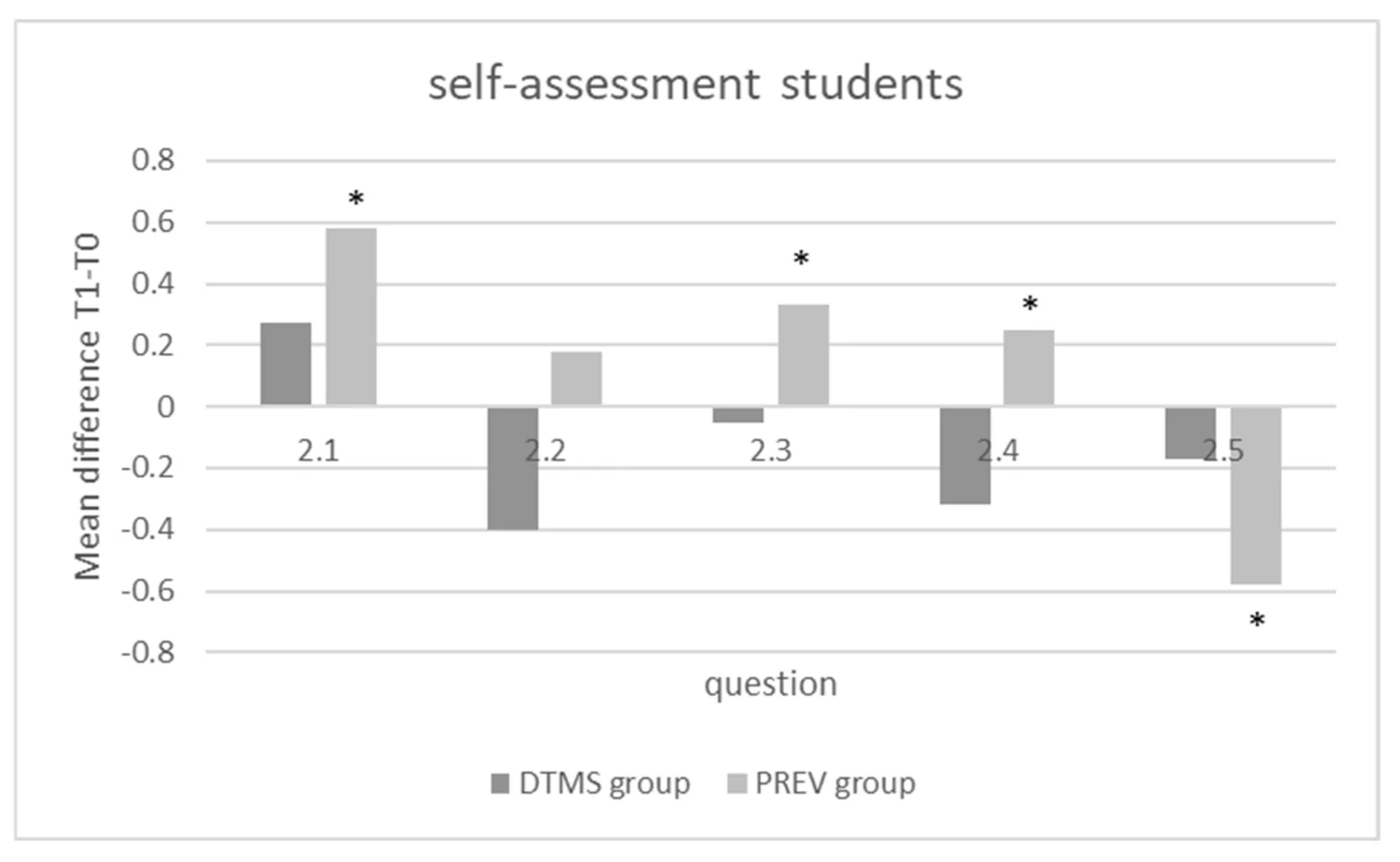
Difference between the mean scores of students’ self-assessments of DTMS and PREV groups at t1 and t0 (* significant at *p* < 0.05).

Students in the PREVG rated questions 2.1 (overall satisfaction with their own preparations, *p* = 0.006), 2.3 (adherence to preparation forms; *p* = 0.011), 2.4 (smoothness of the preparation; *p* = 0.054) and 2.5 (overall grade; *p* < 0.001) significantly higher at time point t1 than at the start of the study. No significant differences were found in the DTMSG for question 2.2 (adherence to preparation depth). Furthermore, the comparison of t0 and t1 revealed no significant differences between the PREVG and the DTMSG (Figure 6).

### 3.7. Comparison of Self-Assessment and External Assessment Grades

In the DTMSG, no significant differences were observed in the mean grades at t0 and t1. This was observed for the students’ self-assessment (*p* = 0.477) and for the raters’ assessment (*p* = 0.168).

**Figure 7:**
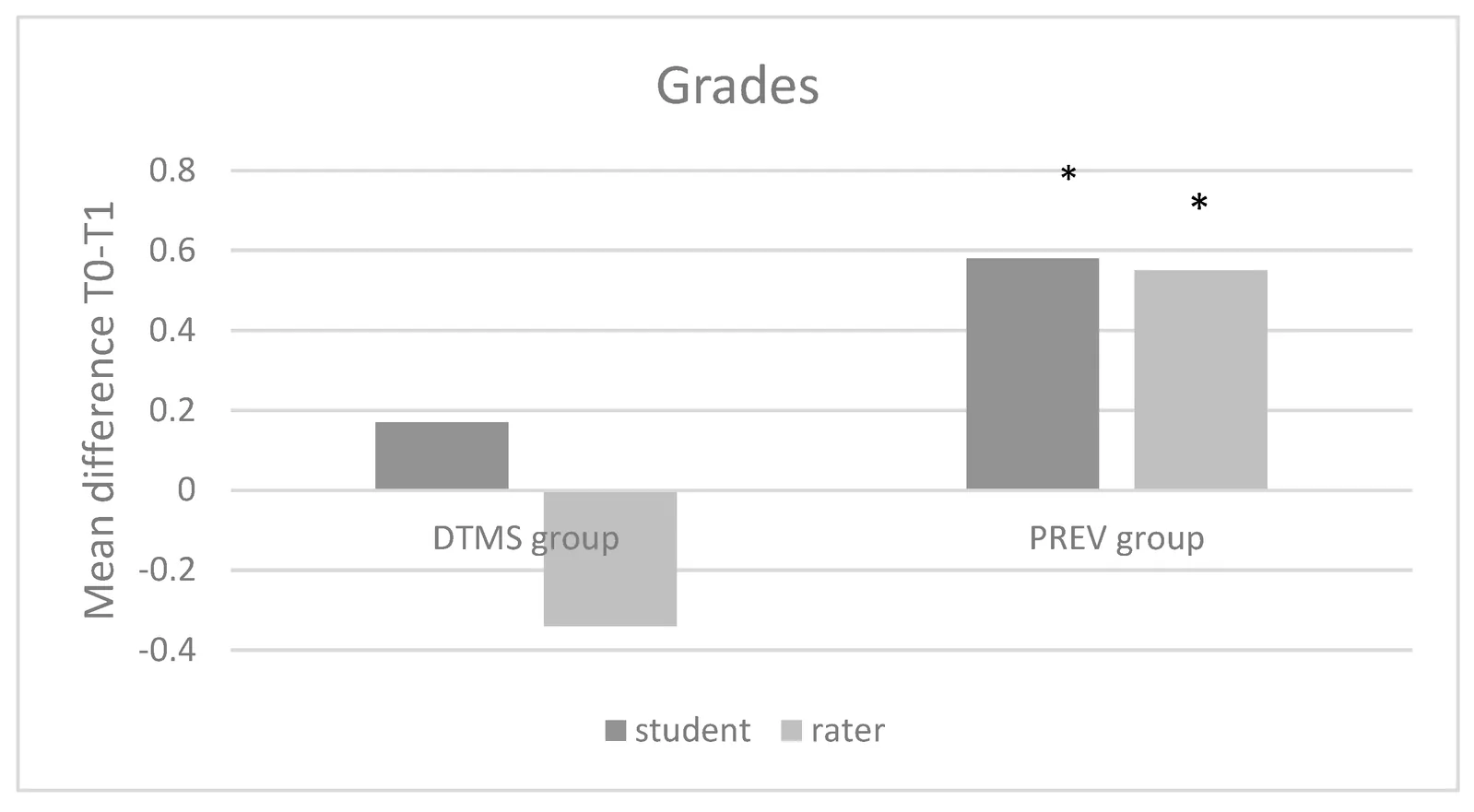
Comparison of the difference between the mean scores of students’ self-assessments and the external assessments by rater (* significant at *p* < 0.05).

In contrast, the PREVG showed significant improvements in both the students’ self-assessment of grades (*p* < 0.001) and the external assessment by the dentists (*p* = 0.002), with grades falling from 3.39 to 2.81 between the two measurement points. There were no significant differences between the DTMSG and the PREVG in the students’ self-assessment, neither at t0 nor at t1. In the external assessment, the PREVG achieved a significantly better grade at both t0 (*p* = 0.036) and t1 (*p* < 0.001) (Figure 7).

## 4. Discussion

The students in the PREV group (PREVG), who had undergone a reduced amount of preclinical dental technology training and a curriculum focused primarily on the dental treatment procedures, showed a clear improvement in their preparation skills as measured by the overall score for practical exercises. This improvement was also evident in their adherence to the required preparation form and in the smoothness of the preparation. The students’ self-assessment is consistent with these results. No significant improvements were detected in the DTMS group (DTMSG). Although the overall score decreased slightly, this change was not statistically significant and should be interpreted with caution, particularly given the relatively small sample size of the DTMS group. These findings suggest that the educational focus of practical training may be more important than its overall duration. This interpretation is consistent with the international survey by Ba-Hattab et al., which highlighted substantial variation in the content and educational focus of practical dental curricula worldwide [14]. Likewise, McGleenon and Morison emphasized that contemporary dental education is increasingly shifting towards competency-based, clinically relevant training aimed at preparing students for patient care [15].

An analysis of the preparation criteria indicates that the depth of preparation initially achieves the highest values of all evaluated criteria in both groups. In both groups, the values are lower at t1. The depth of preparation is a criterion that can be readily assessed using the transparent blocks provided to both groups. However, an analysis of tooth preparations shows that sufficient reduction in hard tooth substance is an extremely challenging preparation criterion for students. Almohefer et al. demonstrated that the occlusal, axial, lingual and buccal reduction in hard tooth substance for the placement of an all-ceramic crown was not satisfactory among dental students [11]. These findings are also reflected in the study by Poon and Smales [16]. When preparing metal-ceramic veneered crowns, students did not sufficiently reduce the cusps as well as the central fissure. Without adequate reduction in tooth structure, it is not possible to place an indirect restoration. This criterion is easy to verify by using the transparent Frasaco preparation trainers, which allow the amount of tooth structure removed to be checked using a millimeter scale. When practicing on dental models, it can be helpful to use technical aids such as scanning the preparations and assessing them using software [17]. The decrease in preparation depth scores at t1 suggests that greater emphasis should be placed on achieving adequate tooth reduction during the shared preclinical course.

The scores for the preparation form were higher in the PREVG than in the DTMSG right from the start. Furthermore, these scores improved significantly in the PREVG at t1. An improvement was also observed in the DTMSG, although it was not significant. These results show that students continuously practice their manual skills through the course and that improvements are detectable. Pérez-Chicharro et al. demonstrate that manual dexterity can be trained and requires continuous practice [10]. In addition, the 3D-printed dental model developed by Lee et al. has shown that there are significant differences in the manual dexterity of dental students, and that these differences correlate with the precision of cavity preparation [15]. Adherence to the preparation form is essential for carrying out the procedure in a minimally invasive and tooth-preserving method [18].

Analysis of the smoothness of the preparation revealed that the PREVG initially had higher values than the DTMSG, but these improved significantly in the PREVG. In the DTMSG, the values remained at a similar level. The study by Almohefer et al. demonstrated satisfactory smoothness of the preparations when preparing model teeth [11]. The smoothness of the Frasaco preparation trainer is a characteristic that is difficult to assess, as there are few concrete evaluation criteria that can be assessed visually. To examine this criterion more closely, consideration could be given to the use of technical aids, such as a scanner [17]. However, a significant improvement is observed only in the PREVG, indicating that this group is better able to refine their preparations.

The preparation criteria examined form part of the standard criteria defined by the FDI World Dental Federation [18]. Adherence to the preparation form is crucial to avoid removing unnecessary tooth structure. A consistent depth appropriate to the indication must be maintained to allow for the placement of indirect restorations. Surface quality is a further criterion for assessing the precision of a preparation. This study is based on these three criteria, as other aspects, such as the assessment of the cavity walls, cannot be evaluated using the Frasaco preparation trainers. To simplify the scoring system, the points used in Habib’s study have been simplified [19].

The students’ self-assessments in the PREVG show a marked improvement in the overall rating, as well as in the assessment of adherence to the preparation form and the smoothness of the preparation. No differences were observed between the two groups in terms of preparation depth. These assessments are consistent with the external evaluation carried out by the three dentists. Gratton et al. also demonstrated that there were no significant differences between self-assessment and visual assessment of tooth preparations by course supervisors [20]. In addition, feedback provided by new technologies, such as a scanner, could also be of interest here. In the study by Schepke et al., the students described this as a useful and effective addition, as it made the assessments even more comparable [17].

The DTMSG’s self-assessment revealed no significant changes. These results are consistent with those of the external evaluation. Compared to the PREVG, students in the DTMSG rated themselves slightly higher at the start of the course. This could be linked to the increased practice time allocated to the DTMSG during the preclinical phase of the course. As a result, students in the DTMSG are more likely to overestimate their own preparation skills [21]. As the course time increases, self-assessment scores for the three preparation criteria decrease. This suggests that the additional practice time, combined with feedback from lecturers, improves the DTMSG’s self-assessment. The study by Ihm et al. demonstrated that feedback helps participants to better assess their own self-perceived competencies [22]. Deeb et al. likewise demonstrated that technical feedback provided by instructors in clinical periodontology improved the accuracy of students’ self-assessment [23]. These findings underline the importance of effective supervision during preclinical training. Accurate self-assessment is an essential skill for recognizing the limits of one’s own abilities in future patient treatment.

The studies by El-Kishawi and Liu et al. demonstrated that personal preferences play a key role in learning processes [24,25]. In general, students in the DTMSG, who had completed a curriculum with a greater practical workload focused on dental technology and material science, achieved lower overall results than those in the PREVG, whose curriculum emphasized prevention-oriented training and focused on dental treatment procedures. Notably, despite the reduced amount of practical training, the PREVG was not inferior at baseline and even demonstrated better preparation results at both the beginning and the end of the shared course. These findings are also reflected in the overall ratings of self-assessment and external assessment in both groups. From an educational perspective, this suggests that preclinical practical training may benefit from a stronger alignment with clinically relevant competencies and patient care. This interpretation is consistent with the international survey by Ba-Hattab et al., which highlighted substantial variation in the educational content of practical dental curricula, and with the review by McGleenon and Morison, who described a continuing shift towards competency-based, patient-centered dental education [14,15]. Nevertheless, continuous training of fine motor skills remains essential to ensure that students enter the clinical phase as well prepared as possible [10].

### Strengths and Limitations

One of the main limitations is the unequal size of the study groups, which may have reduced the statistical power to detect within-group changes, particularly in the smaller DTMS group. Due to differences in the study regulations, it was not possible to influence the group allocation, sample size or gender distribution, which is why the study design must be considered a quasi-experiment. Consequently, subgroup analyses by gender could not be performed. Almohefer et al. reported that female students tend to proceed more cautiously and place the preparation margins more supragingivally, which may have influenced the preparation criteria investigated in the present study [11].

Furthermore, prior manual skills acquired through hobbies, sports, or artistic activities were not assessed and may have influenced the study outcomes. However, both groups had equal access to practical training, as no additional opportunities for unsupervised practice outside the scheduled courses were available.

At the same time, the parallel existence of both curricula provided a unique opportunity for comparison. This situation was only temporarily possible during the transition from the former German dental curriculum, which was more strongly focused on dental technology and materials science, to the new curriculum emphasizing prevention-oriented and clinically relevant dental procedures. As the former curriculum is currently being phased out, no further cohorts will study under this material- and technology-oriented training concept in the future. Furthermore, students enrolled in the new curriculum may have been particularly motivated and committed, as they represented the first cohorts to complete the newly introduced curriculum following the reform of the licensing regulations. This selection bias must be taken into account when interpreting the results. In addition, the study was conducted at a single study site, which may limit the generalizability of the findings. Further studies involving a broader sample of students should therefore be conducted in order to draw general conclusions.

Interrater reliability analysis demonstrated moderate agreement between the evaluators. Although Fleiss’ kappa indicated only moderate agreement, ANOVA revealed no significant differences in ratings between the evaluators over time, suggesting a stable and consistent overall rating behavior throughout the study period. An intra-rater reliability assessment was not performed, as each specimen was scored only once by each examiner. The rating system was simplified to reduce the complexity of the assessment. However, this approach may have limited the detection of subtle differences between preparations.

Future studies could incorporate objective three-dimensional digital assessment methods, such as CAD/CAM-based analyses, alongside visual examiner ratings. As reported by Schepke et al., digital scanning can provide valuable additional feedback for both students and instructors and may further improve the objectivity of the assessment process [17].

The self-assessment questionnaire was developed by experienced dental educators and pilot-tested before implementation; it was not formally psychometrically validated. When assessing students’ self-assessments, it is important to bear in mind that over- and underestimation may come into play. Constructive feedback is essential here to ensure that self-assessments are as nuanced as possible.

## 5. Conclusions

In conclusion, a preclinical curriculum emphasizing preventive dentistry and a focus on dental treatment procedures was not inferior to a more extensive curriculum focused on materials science and dental technology, despite involving fewer practical exercises. Continuous practice remains essential to adequately prepare students for clinical patient care, although the individual amount of training required may vary considerably. At the same time, reducing the overall practical workload may create additional curricular capacity for modern educational priorities such as interdisciplinary teaching.

## Figures and Tables

**Table 1:** Grading scale for the preparation exercises.

Score	Depth	Form	Smoothness
2	3 mm depth	adhered to form	smooth
1	deviation of up to 1 mm	up to 1 deviation	slightly uneven
0	deviation more than 1 mm	more than one deviation	uneven

**Table 2:** Socio-demographic parameters of the students.

Feature	Characteristics	Number (n)
age	years	25.22 years (SD ± 4.78)
gender	male	20
	female divers	440
previous education	medical vocational training	8
	dental vocational training	9
	Dental technician vocational training	6
	vocational training in skilled trades none other	1 39 1

## Data Availability

The datasets and study materials associated with this manuscript are available in the Open Science Framework (OSF) repository: https://osf.io/9d6eq/overview?view_only=9f12e207614942cd93282ff18f49f615 (Accessed on 16 July 2026).

## Abbreviations

The following abbreviations are used in this manuscript:

DTMS	Dental technology and material science curriculum
DTMSG	Dental technology and material science curriculum group
FDI	World Dental Federation
PREV	Prevention-oriented curriculum
PREVG	Prevention-oriented curriculum group
IMPP	Institute for Medical and Pharmaceutical Examination Questions
